# Impacts of Tobacco Stalk Biochar Remediation in Microplastic-Contaminated Soil on Early Rice Growth Indicators and Soil Quality

**DOI:** 10.3390/plants15071132

**Published:** 2026-04-07

**Authors:** Qiong Yang, Suhang Li, Rou Ma, Longcheng Jiang, Jiaojiao Liu, Jiaxin Yao, Ying Liu, Jun Ren, Yang Luo, Yangzhou Xiang, Xuqiang Luo

**Affiliations:** 1 School of Geography and Resources, Guizhou Education University, Guiyang 550018, China; 2 School of Biological Sciences, Guizhou Education University, Guiyang 550018, China

**Keywords:** biochar, microplastic pollution, soil remediation, rice, plant stress response

## Abstract

Microplastic pollution in farmland soils has emerged as a global concern due to its potential to degrade soil health, inhibit crop growth, and enter the food chain. However, effective and environmentally friendly remediation strategies remain limited, particularly regarding the use of biochar to mitigate polyethylene microplastic (PE-MP) stress in agroecosystems. This study investigates whether tobacco stalk biochar (TSB) can alleviate PE-MPs stress in rice seedlings. A two-factor pot experiment was conducted to systematically analyze the responses of soil physicochemical properties, rice growth indicators, and antioxidant enzyme activities to the combined application of varying concentrations of PE-MPs (0, 0.5%, 1%, and 2% (*w*/*w*)) and TSB (0, 3%, 6%, and 9% (*w*/*w*)). The results show that TSB significantly increased soil pH and organic matter content, effectively mitigating the decline in available nitrogen, phosphorus, and potassium caused by PE-MPs (e.g., under the M3B3 treatment, available nitrogen and phosphorus contents increased by 68.7% and 226%, respectively, compared with those under the M3B0 treatment). Under low-concentration PE-MP (0.5%) stress, an appropriate amount of TSB (3%) resulted in the highest rice germination rate, vigor index, and stress tolerance index, while significantly inducing the activities of superoxide dismutase (SOD) and catalase (CAT) to alleviate oxidative damage. However, high-concentration combinations of TSB and PE-MPs exhibited an antagonistic effect. In conclusion, tobacco stalk biochar can synergistically mitigate microplastic stress on rice through multiple pathways, with its remediation effects exhibiting significant dose dependence and interactive complexity. These findings provide a theoretical and technical basis for the ecological remediation of microplastic pollution in farmland.

## 1. Introduction

Microplastics, an emerging class of pollutants ubiquitously detected in global soil ecosystems, have made their environmental fate and ecological risks a frontier focus in environmental science and sustainable agricultural development [1]. Agricultural soils serve as a significant sink for microplastics, with pollution primarily originating from the residue of agricultural plastic films, sewage sludge application, and atmospheric deposition [2]. These polymer particles, smaller than 5 mm in size, not only persist in the soil for extended periods [3] but also pose a profound threat to soil health by altering soil aggregation structure, water conductivity, and nutrient cycling [4]. Recent studies indicate that microplastic accumulation can significantly inhibit soil microbial activity, alter community structure, and impair the function of key soil enzymes, thereby disrupting biogeochemical nutrient cycling processes [5,6]. Of greater concern is the ability of microplastics to act as vectors, adsorbing and co-transporting contaminants such as heavy metals and pesticides, leading to combined pollution and synergistic toxic effects [7,8]. With global plastic production continuing to rise, systematically elucidating the multifaceted negative impacts of microplastics on farmland ecosystems and developing effective remediation technologies have become urgent necessities for safeguarding global food security and ecological safety [9,10].

Biochar, a carbon-rich porous material derived from biomass pyrolysis, has garnered widespread attention for soil improvement and pollution remediation [11,12]. Its mechanisms include physicochemical adsorption of pollutants [13,14], chemical regulation of soil pH and nutrient retention [15,16], and biological effects such as enhancing soil organic matter and providing habitats for beneficial microorganisms [17,18]. Numerous studies have confirmed that biochar can alleviate various abiotic stresses by improving the rhizosphere microenvironment and inducing systemic resistance, thereby promoting crop growth and enhancing antioxidant defense systems [19,20,21].

While substantial research exists separately on microplastic ecotoxicology and biochar soil improvement, their interactive effects within the “soil–pollutant–amendment–plant” system remain understudied [22,23]. The limited preliminary studies reveal complex and potentially contradictory results [24,25,26,27]. Furthermore, non-linear dose–response relationships may exist between biochar properties and microplastic characteristics [28,29,30]. However, most existing research has focused on conventional biochars (e.g., rice husk, corn straw, and wood). The potential of tobacco stalk biochar (TSB), an abundant agricultural waste, has been largely unexplored in microplastic remediation. TSB possesses unique properties that distinguish it from other biochars, including higher potassium content, abundant oxygen-containing functional groups, and a well-developed porous structure due to the fibrous nature of tobacco stalks [31]. These characteristics may confer superior capabilities in alleviating microplastic-induced nutrient limitation and oxidative stress in crops.

Several critical knowledge gaps, therefore, remain. First, the combined effects of TSB and polyethylene microplastics (PE-MPs) on rice (*Oryza sativa* L.) have not been systematically evaluated, despite PE-MPs being one of the most commonly detected microplastic types in agricultural soils and rice being a staple crop, feeding over half the global population. Second, it remains unclear whether high TSB application rates could produce antagonistic or over-remediation effects that counteract its benefits. To address these gaps, this study aims to (1) systematically investigate the remediation potential of TSB in PE-MPs-contaminated soil by analyzing soil physicochemical properties (pH, organic matter, and available N/P/K); (2) evaluate the growth performance of rice seedlings under combined TSB and PE-MPs treatments; and (3) reveal the physiological mechanisms by which TSB regulates antioxidant enzyme activities (SOD and CAT) in rice under microplastic stress.

We propose the following hypotheses: (H1) TSB significantly increases soil pH and organic matter while alleviating PE-MP-induced declines in available nutrients; (H2) appropriate TSB amounts improve rice germination and growth under PE-MP stress while enhancing antioxidant enzyme activities; and (H3) the remediation effects of TSB are dose-dependent, with excessive application potentially leading to antagonistic effects under high-PE-MP stress. The novelty of this study lies in being the first to systematically demonstrate the unique, dose-dependent remediation capacity of tobacco stalk biochar in polyethylene microplastic-contaminated rice systems. This work provides a theoretical and technical basis for using agricultural waste-derived biochar to remediate microplastic-polluted farmlands.

## 2. Materials and Methods

### 2.1. Experimental Materials

The test soil was collected from the typical topsoil (0–20 cm) of a paddy field in Wudang District, Guiyang City, Guizhou Province. Visible stones and plant residues were removed, and the soil was air-dried and passed through a 2 mm nylon sieve for subsequent use. The basic physicochemical properties of the soil were pH 5.96, an organic matter content of 35.08 g kg^−1^, an alkali-hydrolysable nitrogen content of 21.25 mg kg^−1^, an available phosphorus content of 40.78 mg kg^−1^, and an available potassium content of 63.40 mg kg^−1^. The tested tobacco stalk biochar was produced by slow pyrolysis of flue-cured tobacco stalks under oxygen-limited conditions at 380 °C for 2 h (supplied by Guizhou Jinyefeng Agricultural Technology Co., Ltd. (Bijie, China)). Its main characteristics were: pH 9.18; C, H, O, and N contents of 48.30%, 3.93%, 31.40%, and 1.50%, respectively; a total phosphorus content of 2.38%; a total potassium content of 2.44%; an available potassium content of 161.9 mg kg^−1^; a specific surface area of 1.47 m^2^ g^−1^; and an average pore diameter of 20.31 nm [31]. Polyethylene (PE) powder (with a density of 0.92 g cm^−3^) was added as the microplastic treatment to simulate the current status of microplastic pollution in farmland soils [32]. The particles used were pristine (non-aged) spherical particles with an average diameter of approximately 150 μm. The specific surface area (BET) of these pristine spherical particles was determined to be 0.962 m^2^ g^−1^. No further physical or chemical aging treatment was applied to the particles prior to the experiment. The tested rice variety was Zhenghan 10. The experiment used plastic pots (with a top diameter of 17 cm, a bottom diameter of 13 cm, and a height of 16 cm), each filled with a fixed amount of soil. Treatments involving biochar, microplastics, etc., were thoroughly mixed with the soil according to the designed ratios before rice planting.

### 2.2. Experimental Design

The pot experiment was conducted in a plastic greenhouse at Guizhou Education University (106°47′ E, 26°38′ N), Wudang District, Guiyang City, Guizhou Province. A two-factor completely randomized design was adopted. The first factor was polyethylene microplastics (PE-MPs, M), applied at four levels, 0, 0.5%, 1%, and 2% (*w*/*w*), corresponding to 0, 5, 10, and 20 g per pot. While these levels exceed typical background concentrations in most agricultural soils, they were deliberately selected to represent worst-case contamination scenarios (e.g., hotspots with long-term plastic film mulching or waste accumulation) and were consistent with concentrations used in comparable mechanistic studies [33,34]. The second factor was tobacco stalk biochar (TSB, B), applied at four levels, 0, 3%, 6%, and 9% (*w*/*w*), corresponding to 0, 30, 60, and 90 g per pot. These rates, selected based on preliminary experiments and previous pot studies [28,35,36], were higher than typical field recommendations. This design ensured a wide gradient for evaluating dose-dependent effects and enabled the detection of measurable responses in soil properties and plant performance under controlled stress conditions. This resulted in a total of 16 treatment combinations (Table 1), with each treatment replicated three times.

For each treatment, 1000 g of soil was weighed, and the corresponding amounts of tobacco stalk biochar and polyethylene microplastics were added according to the design scheme. After thorough mixing, the mixture was placed into labeled plastic pots. The soil was moistened with water and allowed to equilibrate for two days before sowing. Rice seeds were surface-sterilized with 75% alcohol for 30 min, washed clean with purified water, and soaked for 12 h. Sowing was conducted at a depth of 1–2 cm in each pot. A flooding layer of approximately 2 cm above the soil surface was maintained during the experiment. The germination rate was measured 7 days after sowing. Soil and plant samples were collected 30 days after sowing for subsequent analysis.

### 2.3. Sample Collection and Analysis

#### 2.3.1. Measurement of Growth Parameters

The number of germinated rice seeds was recorded on the 7th day after sowing, and the seedling length was measured using a ruler. The germination parameters measured in this experiment were derived using the following equations [37]:

(1) $$Germination\;rate\;(GR)=\frac{N_{g}}{N_{t}}\times 100$$

(2) Vigour index(VI)=GR×seedling length,

(3) $$Stress\;tolerance\;index\left(STI\right)=\frac{{SL}_{T}}{{SL}_{CK}}\times 100,$$

where N_g_ denotes the number of germinated seeds on the seventh day, while N_t_ signifies the total number of tested seeds. SL_CK_ indicates the shoot length of the control group, and SL_T_ corresponds to the shoot length under the specific treatment.

#### 2.3.2. Measurement of Physiological Parameters

On the 30th day after sowing, the growth and physiological parameters of the rice seedlings were determined, including shoot length (SL, cm), superoxide dismutase activity (SOD, U g^−1^), and catalase activity (CAT, U (g·min)^−1^). The specific determination methods were as follows. (1) Shoot length measurement: The aboveground part height (from the shoot base to the highest leaf tip) of the rice plants was measured using a measuring tape. Twelve representative seedlings per pot were selected for measurement, and their average value represented the shoot length of that pot. (2) SOD activity assay: Determined by the nitro blue tetrazolium (NBT) photoreduction method, as described in reference [38]. (3) CAT activity assay: Determined by the ultraviolet absorption method, as described in reference [39].

#### 2.3.3. Measurement of Soil Physicochemical Properties

Soil physicochemical indicators were determined following the methods described in *Soil and Agricultural Chemistry Analysis* (Third Edition) [40]. The specific procedures were as follows: (1) Soil pH was measured using the water extraction potentiometric method (water-to-soil ratio of 2.5:1). (2) The organic matter content was determined using the potassium dichromate external heating method. (3) The alkali-hydrolysable nitrogen content was measured using the alkaline hydrolysis diffusion method. (4) The available phosphorus content was determined using ammonium fluoride–hydrochloric acid extraction, followed by the molybdenum–antimony colorimetric method. (5) The available potassium content was measured using the ammonium acetate extraction–flame photometry method.

### 2.4. Data Analysis

Experimental data were preliminarily processed using Excel 2016, and the results were expressed as means ± standard deviation (n = 3). Statistical analysis was performed using the SPSS software (version 26.0). Before conducting two-way ANOVA, the assumptions of normality and homogeneity of variances were tested using the Shapiro–Wilk test and Levene‘s test, respectively. All residuals met the assumptions (*p* > 0.05). Two-way ANOVA was then used to systematically evaluate the main effects and interaction effects of the two factors, tobacco stalk biochar (TSB) and polyethylene microplastics (PE-MPs), on rice seedling growth indicators, physiological characteristics, and soil properties. When significant differences were detected in the two-way ANOVA, simple effect analyses were conducted for significant interactions, and post hoc multiple comparisons were performed using the least-significant difference (LSD) method, with significance levels adjusted for multiple comparisons where appropriate. Graphs were generated using Origin 2026.

## 3. Results

### 3.1. Effects of Different Treatments on Soil pH and Organic Matter

**Figure 1:**
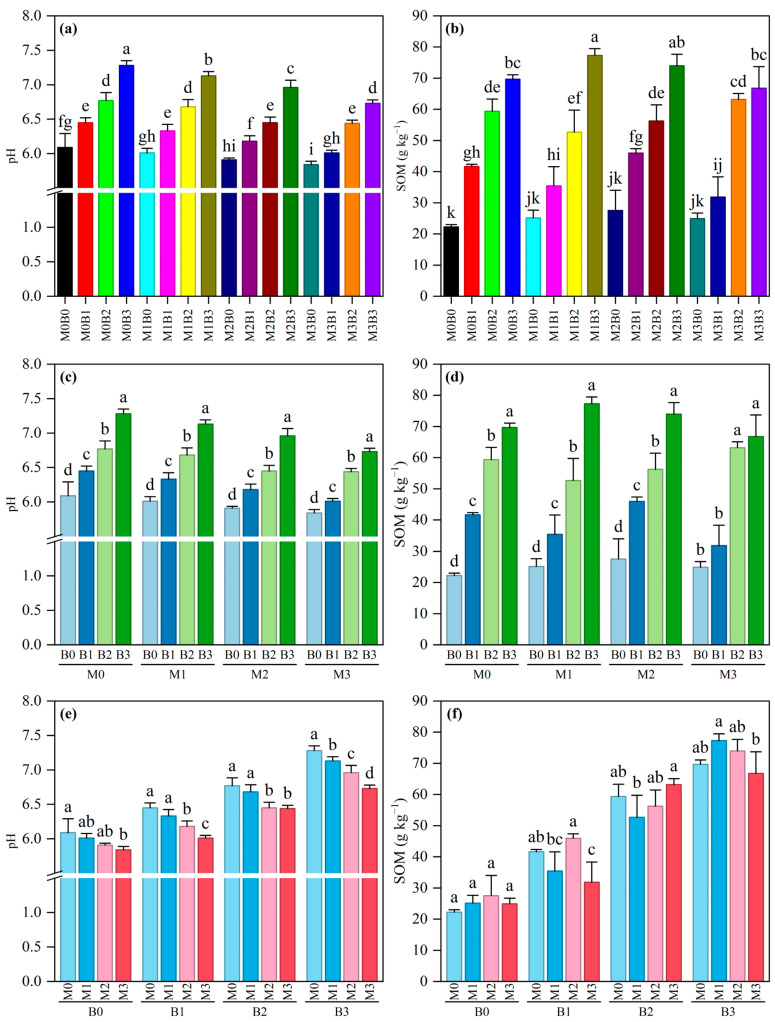
Impact of TSB on soil pH and organic matter (SOM) in PE-MP-contaminated conditions. Error bars represent ± standard deviation (n = 3). (**a**) Combined Effects of PE-MP and Biochar on soil pH; (**b**) Combined Effects of PE-MP and Biochar on SOM; (**c**) Impact of different TSB on soil pH at the same PE-MP concentration; (**d**) Impact of different TSB on SOM at the same PE-MP concentration; (**e**) Impact of different PE-MP concentrations on soil pH at the same TSB; (**f**) Impact of different PE-MP concentrations on SOM at the same TSB. Different lowercase letters indicate significant differences among treatments at the 0.05 level.

Using two-way analysis of variance and multiple comparisons, this experiment systematically investigated the effects of polyethylene microplastics (PE-MPs), tobacco stalk biochar (TSB), and their interaction on soil pH and soil organic matter (SOM) content (Table S1). The results showed that both PE-MPs and TSB significantly affected soil pH and SOM, but their interactive effects differed. For soil pH, the main effects of both PE-MPs and TSB were highly significant (*p* < 0.001), while their interaction was not significant (*p* = 0.213). Specifically (Figure 1a,c,e), under the same PE-MP stress level, the soil pH increased significantly with an increasing TSB application rate (from B0 to B3). For example, under the M0 condition, the pH increased from 6.09 in B0 to 7.28 in B3. Conversely, at the same TSB level, the soil pH exhibited a decreasing trend with an increasing PE-MPs concentration (from M0 to M3). Taking the B3 treatment as an example, the pH decreased significantly from 7.28 in M0 to 6.73 in M3. These results indicate that TSB can effectively increase soil pH and buffer soil acidity, while the addition of PE-MPs induces soil acidification.

Regarding soil organic matter (SOM), the main effect of TSB was highly significant (*p* < 0.001), while the main effect of PE-MPs was not significant (*p* = 0.117); however, their interaction was significant (*p* = 0.002). As shown in Figure 1b,d,f, under different PE-MPs levels, the SOM content increased significantly with an increasing TSB application rate, highlighting the core role of biochar as an organic carbon source. The interactive effect was evident as the variation in the PE-MP concentration altered the enhancement pattern of TSB. For instance, under M1 stress, the SOM content increased sharply from 25.13 in B0 to 77.31 in B3, whereas under no-stress (M0) or high-stress (M3) conditions, the increment pattern of the TSB treatments differed. This suggests that the presence of microplastics may regulate the organic matter accumulation process by affecting the soil micro-environment or the function of biochar.

### 3.2. Effects of Different Treatments on Soil Available Nutrients

**Figure 2:**
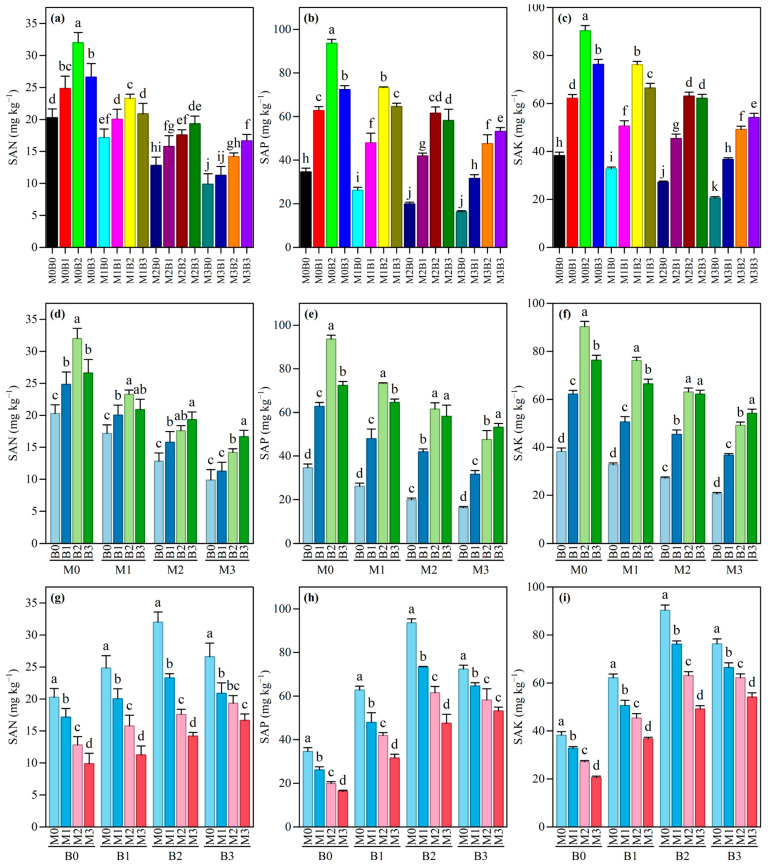
Impact of TSB on soil available nutrients under PE-MP contamination. Error bars denote standard deviations (n = 3). (**a**) Combined Effects of PE-MP and TSB on SAN; (**b**) Combined Effects of PE-MP and TSB on SAP; (**c**) Combined Effects of PE-MP and TSB on SAK; (**d**) Impact of different TSB on SAN at the same PE-MP concentration; (**e**) Impact of different TSB on SAP at the same PE-MP concentration; (**f**) Impact of different TSB on SAK at the same PE-MP concentration; (**g**) Impact of different PE-MP concentrations on SAN at the same TSB; (**h**) Impact of different PE-MP concentrations on SAP at the same TSB; (**i**) Impact of different PE-MP concentrations on SAK at the same TSB. Different lowercase letters indicate significant differences among treatments at the 0.05 level.

The contents of soil available nutrients were significantly affected by the treatments of polyethylene microplastics (PE-MPs) and tobacco stalk biochar (TSB). The results of two-way analysis of variance indicated that PE-MPs, TSB, and their interaction (PE-MPs × TSB) had highly significant effects on all three available nutrient indicators (soil available nitrogen [SAN], soil available phosphorus [SAP], and soil available potassium [SAK]) (*p* < 0.001) (Table S2). The specific performances were as follows: For soil available nitrogen (SAN), two-way ANOVA revealed that PE-MPs (*F* = 184.346), TSB (*F* = 55.379), and their interaction (*F* = 4.768) all exerted highly significant effects on SAN content (Table S2). The data showed that PE-MPs exerted a significant dose-dependent inhibitory effect on SAN. In the absence of biochar (B0), the SAN content decreased significantly from 20.29 mg kg^−1^ to 9.87 mg kg^−1^ as the PE-MP concentration increased from M0 to M3 (Figure 2g). The addition of TSB effectively alleviated this inhibition; at the same PE-MPs level (e.g., M1), the SAN content increased significantly with an increasing biochar application rate (from 17.17 mg kg^−1^ in B0 to 23.29 mg kg^−1^ in B2) (Figure 2d). Notably, the significant interaction indicated that the mitigating effect of biochar depended on the stress level of PE-MPs. Under the high-concentration PE-MP condition (M3), the B3 treatment increased SAN by 68.7% compared with the B0 treatment, highlighting the improvement potential of biochar under adverse conditions.

For soil available phosphorus (SAP), ANOVA revealed that the effects of PE-MPs (*F* = 296.114), TSB (*F* = 788.951), and their interaction (*F* = 14.745) all reached highly significant levels (Table S2). The addition of PE-MPs led to a systematic decrease in the SAP content. For example, under the B1 condition, increasing the PE-MP concentration from M0 to M3 decreased SAP from 62.78 mg kg^−1^ to 31.61 mg kg^−1^ (Figure 2h). However, TSB exhibited a strong enhancing effect, particularly under no PE-MP stress, where the SAP content in the M0B2 treatment (93.62 mg kg^−1^) was significantly higher than that in the other treatments (Figure 2e). The significant interaction further elucidated that the enhancing effect of biochar on SAP was modulated under PE-MP stress. Nevertheless, even under the highest concentration of PE-MPs (M3), the application of high-rate biochar (B3) still achieved an SAP content of 53.20 mg kg^−1^, significantly higher than the control without biochar (16.32 mg kg^−1^) (Figure 2e), demonstrating the outstanding contribution of biochar to mitigating phosphorus availability.

The soil available potassium (SAK) content was highly significantly affected by PE-MPs (*F* = 628.382), TSB (*F* = 1623.778), and their interaction (*F* = 25.442) (Table S2), with the main effect of TSB having the highest *F*-value, indicating that biochar was the strongest factor regulating soil available potassium. In the absence of PE-MPs, the SAK value in the M0B2 treatment was the highest (90.30 mg kg^−1^) (Figure 2f). PE-MP stress significantly reduced SAK, but at any given PE-MP level, increasing the biochar application rate significantly reversed this decreasing trend (Figure 2f). The significant interaction indicated that PE-MPs and TSB jointly determined the final level of SAK. For instance, under M2 stress, the B2 treatment resulted in a SAK content (63.11 mg kg^−1^) that was significantly higher than that of the B0 treatment (27.22 mg kg^−1^) (Figure 2f). At each biochar application level, SAK showed a progressive decrease with an increasing PE-MPs concentration (Figure 2i); however, the addition of biochar significantly elevated the baseline level in each case, effectively buffering the negative effects of microplastics.

### 3.3. Effects of Different Treatments on Rice Seedling Growth

**Figure 3:**
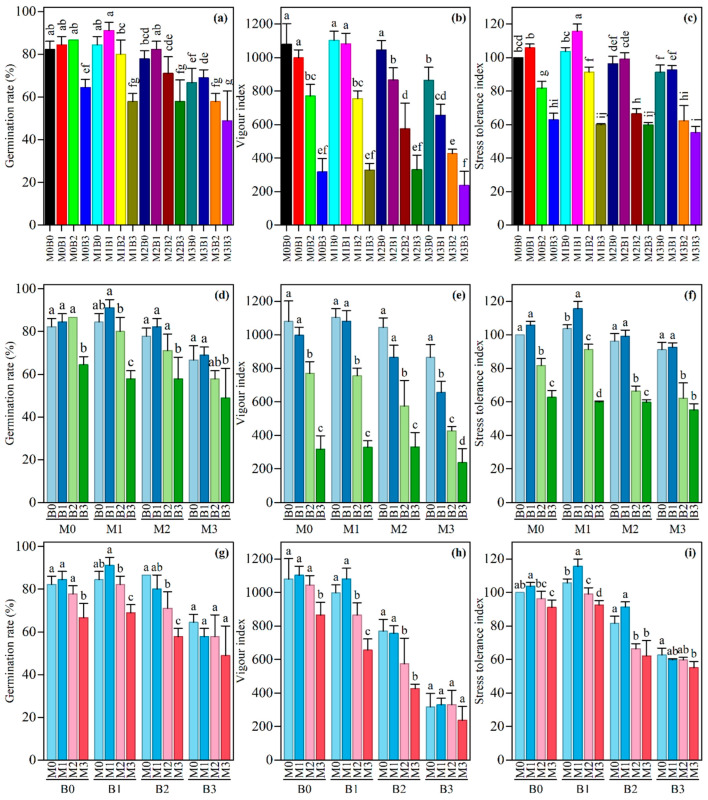
Effect of TSB on growth parameters of rice seedling under PE-MP contamination. Error bars represent ± standard deviation (n = 3). (**a**) Combined Effects of PE-MP and TSB on germination rate; (**b**) Combined Effects of PE-MP and TSB on vigor index; (**c**) Combined Effects of PE-MP and TSB on stress tolerance index; (**d**) Impact of different TSB on germination rate at the same PE-MP concentration; (**e**) Impact of different TSB on vigor index at the same PE-MP concentration; (**f**) Impact of different TSB on stress tolerance index at the same PE-MP concentration; (**g**) Impact of different PE-MP concentrations on germination rate at the same TSB; (**h**) Impact of different PE-MP concentrations on vigor index at the same TSB; (**i**) Impact of different PE-MP concentrations on stress tolerance index at the same TSB. Different lowercase letters indicate significant differences among treatments at the 0.05 level.

Under microplastic (PE-MP) stress, the germination rate, vigor index, and stress tolerance index of rice seedlings were significantly affected. As shown in Figure 3a, among the different combinations of microplastics and tobacco stalk biochar (TSB) treatments, the M1B1 treatment had the highest germination rate (91.11% ± 3.85), significantly higher than most combinations, whereas the M3B3 treatment showed the lowest germination rate (48.89% ± 13.88), indicating that the combination of high-concentration microplastics and a high biochar rate exacerbated the inhibition of germination. Regarding the vigor index (Figure 3b), the M1B0 and M1B1 treatments maintained relatively high levels (1102.40 ± 54.15 and 1081.09 ± 63.65, respectively), significantly outperforming other treatments; in contrast, the M3B3 treatment had the lowest vigor index (236.67 ± 84.20). The results of the stress tolerance index (Figure 3c) further revealed that the M1B1 treatment had the highest stress tolerance (115.55 ± 4.43), significantly higher than the control group and other treatment combinations, while the M3B3 treatment showed the lowest tolerance (55.157 ± 3.705).

Regarding interactions, at the same microplastic level (Figure 3d–f), an appropriate amount of biochar (B1) generally alleviated stress, particularly under the M1 condition, where it enhanced all three indicators; however, a high biochar rate (B3) significantly reduced the values of each indicator. At the same biochar level (Figure 3g–i), with an increasing microplastic concentration, the germination rate, vigor index, and stress tolerance index all gradually decreased, especially under the B3 condition, where the M3 treatment showed the lowest values for all indicators among all groups. Two-way analysis of variance (Table S3) indicated that the main effects of microplastics and biochar on the three indicators were all highly significant (*p* < 0.001). The interaction between the two factors had significant effects on the vigor index (*p* = 0.029) and the stress tolerance index (*p* < 0.001), but no significant interactive effect on the germination rate (*p* = 0.303), suggesting complex synergistic or antagonistic interactions between microplastics and biochar during seedling growth and stress response processes.

### 3.4. Effects of Different Treatments on SOD and CAT Activities in Rice Seedlings

**Figure 4:**
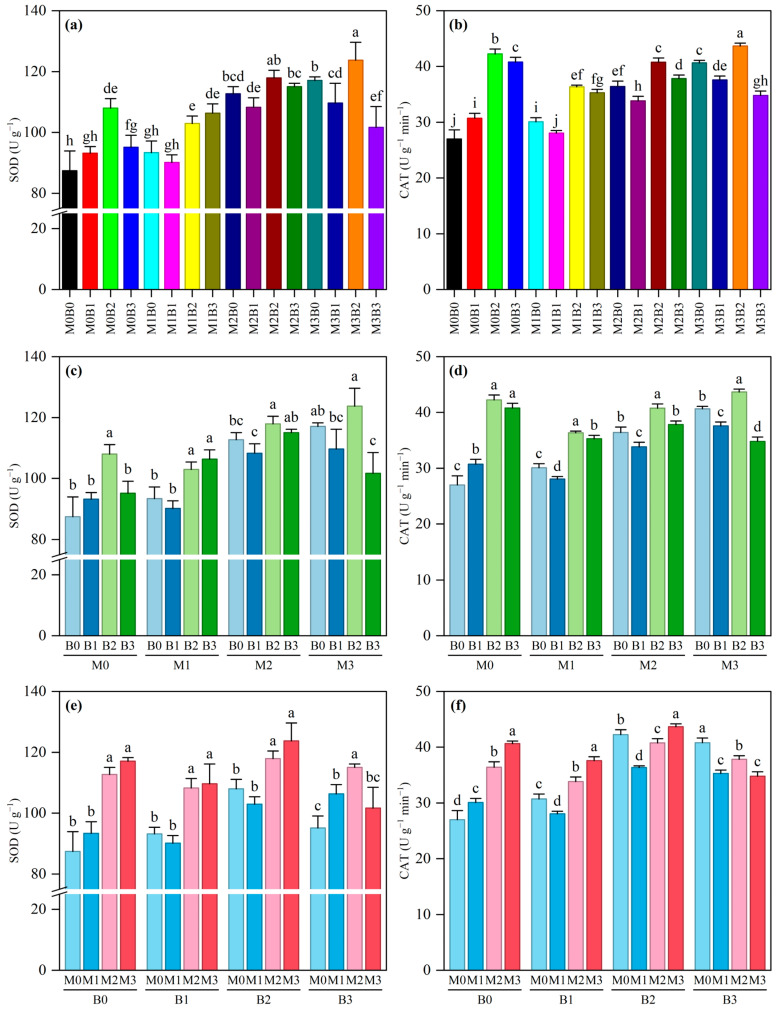
Influence of TSB on the physiological properties of rice seedlings exposed to PE-MP contamination. Error bars indicate the standard deviation (n = 3). (**a**) Combined Effects of PE-MP and TSB on SOD; (**b**) Combined Effects of PE-MP and TSB on CAT; (**c**) Impact of different TSB on SOD at the same PE-MP concentration; (**d**) Impact of different TSB on CAT at the same PE-MP concentration; (**e**) Impact of different PE-MP concentrations on SOD at the same TSB; (**f**) Impact of different PE-MP concentrations on CAT at the same TSB. Different lowercase letters indicate significant differences among treatments at the 0.05 level.

Under microplastic (PE-MP) stress, the addition of varying concentrations of tobacco stalk biochar (TSB) significantly affected the antioxidant enzyme activities of rice seedlings. The changes in superoxide dismutase (SOD) and catalase (CAT) activities are presented in Figure 4 and Table S4. One-way analysis of variance indicated that SOD and CAT activities differed significantly among the treatment combinations (Figure 4a,b). The SOD activity was highest (123.77 U·g^−1^) under the M3B2 treatment, significantly higher than under the control group, M0B0 (87.45 U·g^−1^). The CAT activity was also highest under the M3B2 treatment (43.67 U·g^−1^), significantly higher than under the control group, M0B0 (27.01 U·g^−1^). Analysis of the two-way interaction further revealed that, at the same microplastic concentration, appropriate biochar addition (especially the B2 level) significantly enhanced SOD and CAT activities (Figure 4c,d), whereas at the same biochar level, both enzyme activities generally increased with an increasing microplastic concentration (Figure 4e,f), indicating that the antioxidant system was activated to cope with oxidative stress.

Two-way analysis of variance confirmed that microplastics (PE-MPs), biochar (TSB), and their interaction (PE-MPs × TSB) all significantly affected SOD and CAT activities (*p* < 0.001) (Table S4). Among these, the main effects of microplastics on SOD (*F* = 66.453) and CAT (*F* = 153.671) were extremely significant; the effects of biochar were also highly significant (SOD: *F* = 23.563; CAT: *F* = 260.011). The interaction terms were also significant (SOD: *F* = 7.264; CAT: *F* = 60.577), indicating that biochar can modulate the physiological response of rice to microplastic stress and alleviate oxidative damage.

## 4. Discussion

### 4.1. Biochar-Mediated Amelioration of Soil Properties Under Microplastic Stress

Notably, the tobacco stalk biochar used in this study had a low specific surface area (1.47 m^2^ g^−1^), consistent with its low-temperature pyrolysis without activation. Given this, the observed remediation effects are unlikely to be primarily driven by physical adsorption. These effects include increased soil pH, enhanced nutrient availability, and alleviated oxidative stress. Instead, they are more likely attributable to alkalinity-induced pH buffering [15], direct nutrient supply [41,42], and modulation of the rhizosphere microbial community [43,44].

Through systematic experiments, this study empirically tested the three hypotheses proposed in the introduction. First, the hypothesis that “biochar can significantly increase soil pH and organic matter content in microplastic-contaminated soils, while alleviating the decline in available nutrients induced by microplastics” was fully confirmed. The results demonstrated that the addition of tobacco stalk biochar (TSB) significantly elevated soil pH, with a dose-dependent increase across the treatment gradient from no biochar (B0) to high-rate biochar (B3). The mechanism underlying biochar’s pH-elevating effect is directly related to its alkaline nature; fresh biochar typically contains alkaline components such as carbonates and oxides, which are slowly released into the soil, neutralizing soil acidity [15]. Concurrently, the abundant oxygen-containing functional groups on the biochar surface (e.g., carboxyl and phenolic hydroxyl groups) exhibit strong proton exchange capacity, further enhancing its regulatory effect on soil pH [45]. This pH-elevating effect is particularly critical in microplastic-contaminated soils, as microplastics may release organic acids during aging processes, exacerbating soil acidification [46]. This study found that even under the highest concentration of microplastic stress (M3), the addition of 9% biochar kept soil pH at a pH of 6.73, significantly higher than the control. This provides crucial evidence for remediating microplastic pollution in acidic soil regions.

Changes in soil organic matter (SOM) content further validated the research hypothesis. The data showed that under different microplastic pollution levels, the SOM content increased significantly with an increasing biochar application rate, primarily attributable to biochar itself serving as a stable aromatic carbon source that directly contributes to the soil carbon pool [47,48]. Notably, under M1 stress, SOM increased sharply from 25.13 g kg^−1^ in B0 to 77.31 g kg^−1^ in B3, an increase of 208%, suggesting that mild microplastic pollution may enhance the synergistic effect between biochar and native soil organic matter. This synergistic effect may be related to microplastic-induced alterations in soil aggregation structure. Microplastic particles may act as nuclei promoting the formation of organo–mineral complexes, and biochar addition further enhances this aggregation effect [49]. From the perspective of carbon cycling, biochar addition not only increases total soil organic carbon content but, more importantly, alters the stability of carbon fractions. The aromatic carbon structures in biochar are highly resistant to decomposition, with mean residence times in soil potentially reaching hundreds of years, which is crucial for building a stable soil carbon pool and enhancing soil carbon sequestration capacity [18,50].

Regarding available nutrients, biochar effectively alleviated the decline in available nitrogen, phosphorus, and potassium contents induced by polyethylene microplastics. For soil available nitrogen (SAN), this study revealed that PE-MPs exhibited a significant dose-dependent inhibitory effect, which may be related to the influence of microplastics on soil nitrification–denitrification processes [51]. Microplastic addition may alter soil pore structure and affect oxygen diffusion, thereby inhibiting ammonia-oxidizing bacteria activity and reducing nitrification [52]. Biochar addition may alleviate this inhibition by improving soil aeration and providing microbial habitats [24]. For soil available phosphorus (SAP), biochar demonstrated a strong enhancing effect, closely linked to biochar’s specific adsorption capacity for phosphates. Metal oxides on the biochar surface (e.g., Fe, Al, and Ca oxides) can form inner-sphere complexes with phosphate ions, reducing phosphorus fixation and enhancing its availability [53]. Regarding soil available potassium (SAK), biochar itself is rich in potassium; the tobacco stalk biochar used in this study had an available potassium content of 161.9 mg kg^−1^, directly supplementing the soil potassium pool. Simultaneously, biochar enhanced the soil’s potassium retention capacity by improving cation exchange capacity [41,42]. These multifaceted amelioration mechanisms collectively contributed to a comprehensive validation of research hypothesis H1.

In addition to the direct amelioration of soil physicochemical properties, the interaction between TSB and PE-MPs may involve alterations in the environmental behavior of microplastics themselves. Biochar’s porous structure and abundant surface functional groups enable the adsorption of microplastic particles, potentially reducing their mobility, bioavailability, and physical contact with root surfaces [54,55]. This immobilization effect may represent an initial step in mitigating microplastic-induced stress, thereby enhancing the subsequent positive effects of biochar on soil nutrient retention and plant growth.

### 4.2. Physiological Pathways of Biochar in Alleviating Microplastic Phytotoxicity

The hypothesis that “appropriate amounts of biochar can effectively improve the germination and growth of rice seedlings under microplastic stress, while enhancing their antioxidant enzyme activities” was clearly supported by this study. During the rice germination stage, microplastic stress significantly inhibited seed germination, which may be due to multiple factors: first, microplastic particles may physically hinder seed–soil contact, affecting water absorption [56]; second, additives released from microplastics (e.g., plasticizers and stabilizers) may exert phytotoxic effects [57]; and third, microplastics alter soil microbial communities, thereby affecting the promoting effects of beneficial microorganisms on seed germination [15]. The addition of an appropriate amount of biochar (B1) effectively alleviated these negative effects, with the germination rate reaching 91.11% in the M1B1 treatment, significantly higher than the rates in other treatment combinations. The alleviation mechanisms of biochar may include reducing the bioavailability of microplastics and their released substances through adsorption [54,55]; improving soil pore structure and increasing oxygen supply around seeds [58,59]; and providing a microenvironment conducive to the growth of beneficial microorganisms [43,60].

During the seedling growth stage, this study observed complex dose–response relationships. Under mild microplastic pollution (M1) conditions, an appropriate amount of biochar (B1) maintained the vigor index at a high level of 1081.09, indicating that the promoting effect of biochar was most pronounced. This promoting effect may be related to biochar’s improvement in rhizosphere nutrient status—the rhizosphere is a core region for plant–soil–microorganism interactions, and biochar creates a microenvironment more favorable for root growth by increasing rhizosphere pH, enhancing nutrient availability, and regulating microbial communities [61]. However, with the combination of high-concentration microplastics (M3) and high-rate biochar (B3), significant growth inhibition was observed, with the germination rate dropping to 48.89% and a vigor index of only 236.67. This over-remediation phenomenon may involve multiple mechanisms: excessive biochar can lead to soil over-alkalization, which can affect the availability of trace elements [13]; the synergistic effects between biochar and microplastics may alter soil water characteristics, and in turn, affect root water uptake [62]; and, moreover, high concentrations of exogenous carbon input could trigger microbial priming effects [63], resulting in intensified nutrient competition. These findings highlight the importance of precise dosage control for remediation agents.

The response of the antioxidant enzyme system offers important insights into the mitigation mechanisms of biochar. Microplastic stress significantly increased SOD and CAT activities in rice seedlings, which is a typical response of plants to oxidative stress. Microplastics may induce reactive oxygen species (ROS) production through multiple pathways: by causing direct physical damage to cell membranes [64,65]; by affecting the mitochondrial electron transport chain [66]; and by altering antioxidant synthesis pathways [67]. The addition of biochar further enhanced antioxidant enzyme activities, with a peak in the M3B2 treatment. This enhancing effect may involve multiple pathways: polyphenolic compounds in biochar may exert direct antioxidant effects [68]; biochar improves soil nutrient status, providing sufficient substrates for antioxidant enzyme synthesis [69]; and biochar regulates plant hormone balance, particularly increasing abscisic acid and jasmonic acid levels, thereby activating defense responses [70,71]. Additionally, the regulatory effect of biochar on rhizosphere microbial communities should not be overlooked. Specific microbial taxa may enhance overall plant stress resistance by producing antioxidant substances or by inducing systemic resistance in the plant [44,72].

Furthermore, the regulation of rhizosphere microbial communities may constitute an additional pathway through which biochar alleviates microplastic phytotoxicity. Microplastics have been shown to alter soil microbial community structure and suppress beneficial microbial taxa involved in nutrient cycling and plant growth promotion [6,24]. Biochar, by providing a favorable habitat and carbon source, can restore microbial diversity and activity, thereby indirectly enhancing plant stress tolerance [43,44]. This microbial-mediated mechanism may operate in parallel with the direct physiological effects observed in antioxidant enzyme activities, collectively contributing to the improved growth performance of rice seedlings under combined TSB and PE-MPs treatments.

### 4.3. Integrated Mechanisms and Dose-Dependent Interactions

Synthesizing the findings from soil and plant levels, this study reveals that TSB alleviates PE-MP stress in rice seedlings through four interconnected mechanisms that operate synergistically: (i) soil physicochemical regulation (pH neutralization and organic carbon supplementation); (ii) rhizosphere nutrient availability enhancement (improved retention and cycling of N, P, and K); (iii) plant physiological modulation (priming of antioxidant defense systems); and (iv) biochar–microplastic physical interactions (adsorption and immobilization of microplastic particles). The soil physicochemical regulation observed here aligns with evidence that biochar’s alkaline nature and surface functional groups effectively buffer microplastic-induced acidification [15,45,46], while its stable aromatic carbon directly contributes to soil organic matter pools and carbon sequestration [18,48,50]. Regarding nutrient availability, biochar’s high cation exchange capacity enhances potassium retention [41,42], metal oxides on its surface reduce phosphorus fixation [43,53], and improved soil aeration alleviates microplastic-induced inhibition of nitrification [24,51,52]. For plant physiological modulation, biochar primes antioxidant defense systems through direct radical scavenging by polyphenolic compounds [68], improved nutrient supply for enzyme synthesis [69], and modulation of phytohormone signaling pathways [70,71]. Additionally, biochar’s porous structure facilitates the adsorption and immobilization of microplastic particles, reducing their mobility, bioavailability, and physical damage to root tissues [54,55], an effect particularly important during early plant establishment [56].

A critical insight emerging from this study is the dose-dependent nature of these mechanisms. While moderate biochar application (3–6%) consistently alleviated microplastic stress, the combination of high-concentration PE-MPs (2%) and high-rate biochar (9%) exhibited antagonistic effects, as evidenced by reduced germination rates, vigor indices, and stress tolerance indices (Figure 3). This non-linear response may be attributed to several factors: excessive biochar can lead to over-alkalization, potentially limiting micronutrient availability [13]; high carbon inputs may trigger microbial priming effects, intensifying nutrient competition [63]; and the combined application of high doses of both amendments may alter soil water characteristics, affecting root water uptake [62]. Such antagonistic interactions at high application rates have been increasingly recognized in recent studies examining combined biochar and microplastic effects [26,27].

The significant interaction terms in two-way ANOVA for most soil and plant indicators (Tables S1–S4) further underscore that the effects of TSB and PE-MPs are not simply additive but involve complex synergistic or antagonistic interactions. Notably, the interaction was non-significant for soil pH (*p* = 0.213) but highly significant for available nutrients and antioxidant enzyme activities (*p* < 0.001), suggesting that while biochar’s pH-buffering capacity is robust across different microplastic stress levels, its regulatory effects on nutrient dynamics and plant physiology are more sensitive to the specific combinations of the two factors. This differential interaction pattern highlights the importance of considering both main effects and interactions when evaluating remediation strategies for combined pollution scenarios [22,30].

### 4.4. Limitations and Future Research

Several limitations of this study should be acknowledged. First, the biochar application rates used (3–9%, *w*/*w*) exceed typical field recommendations (1–5%, *w*/*w*), which may alter soil physical properties (e.g., bulk density, porosity, and water retention capacity) beyond agronomically relevant ranges [62,63]. Second, the microplastics were pristine spherical particles, whereas environmentally weathered microplastics exhibit irregular morphologies and surface oxidation that can alter their adsorption capacity and interactions with biochar. Third, the experimental duration was relatively short, precluding assessment of long-term interactions or aging processes between biochar and microplastics under field conditions. Fourth, only polyethylene microplastics were tested, leaving other polymer types and combined pollution scenarios unexplored. Fifth, the mechanistic analysis relied primarily on phenotypic indicators (e.g., shoot length, germination rate) and did not include direct measurements of root morphology, plant tissue nutrient content, or soil microbial communities.

To address these gaps, future research should focus on the following priorities. First, field-scale validation using agronomically relevant biochar rates is needed to determine whether the observed remediation effects are reproducible under practical application conditions. Second, systematic comparisons between pristine and aged microplastics, with special attention to irregular shapes and surface-oxidized particles, should be conducted to validate the generalizability of biochar-based remediation. Third, long-term field experiments are required to evaluate the sustained effects of biochar and microplastics on key soil functions and to capture aging dynamics. Fourth, a broader range of microplastic types and combined pollution systems should be incorporated to assess the scope and constraints of biochar remediation. Fifth, multi-omics technologies should be applied to unravel the underlying mechanisms from the perspectives of rhizosphere microbial regulation and plant molecular responses.

## 5. Conclusions

This study demonstrated that tobacco stalk biochar can alleviate polyethylene microplastic-induced stress in rice seedlings and improve contaminated soil quality. The observed effects were associated with increased soil pH, organic matter, available nutrients (N, P, and K), and enhanced antioxidant enzyme activities (SOD and CAT). The remediation effect was dose-dependent, with 3% biochar + 0.5% microplastics being optimal, while 9% biochar + 2% microplastics showed antagonistic effects. However, direct mechanistic evidence, including root morphology, plant nutrient uptake, and soil microbial community analyses, is currently lacking. The proposed mechanisms should, therefore, be interpreted as working hypotheses pending further validation. Future long-term field studies using multi-omics approaches are needed to clarify the environmental behavior and ecological risks of biochar–microplastic interactions.

## Figures and Tables

**Table 1:** Treatment Schedule.

Treatments	Microplastic Amount (g)	Biochar Amount (g)	Microplastic Ratio (%)	Biochar Ratio (%)
M0B0	0	0	0	0
M0B1	0	30	0	3
M0B2	0	60	0	6
M0B3	0	90	0	9
M1B0	5	0	0.5	0
M1B1	5	30	0.5	3
M1B2	5	60	0.5	6
M1B3	5	90	0.5	9
M2B0	10	0	1	0
M2B1	10	30	1	3
M2B2	10	60	1	6
M2B3	10	90	1	9
M3B0	20	0	2	0
M3B1	20	30	2	3
M3B2	20	60	2	6
M3B3	20	90	2	9

## Data Availability

The original contributions presented in this study are included in this article. Further inquiries can be directed to the corresponding author.

## Supplementary Materials

The following supporting information can be downloaded at https://www.mdpi.com/article/10.3390/plants15071132/s1. Table S1. Results of two-way ANOVA on soil pH and organic matter (SOM) in response to TSB and PE-MPs. “PE-MPs × TSB” denotes the interaction effect between PE-MPs and TSB. Table S2. Results of two-way ANOVA on soil available nutrients in response to TSB and PE-MPs. “PE-MPs × TSB” denotes the interaction effect between PE-MPs and TSB. Table S3. Results of two-way ANOVA on growth parameters of rice seedling in response to TSB and PE-MPs. “PE-MPs × TSB” denotes the interaction effect between PE-MPs and TSB. Table S4. Results of two-way ANOVA on physiological properties of rice seedling in response to TSB and PE-MPs. “PE-MPs × TSB” denotes the interaction effect between PE-MPs and TSB.
