# Supplementary material for: Impacts of Tobacco Stalk Biochar Remediation in Microplastic-Contaminated Soil on Early Rice Growth Indicators and Soil Quality
Source: Plants (Basel). 2026 Apr 7;15(7):1132. doi: 10.3390/plants15071132 (PMC13074882; doi:10.3390/plants15071132)
Supplement: Supplementary file 1 [file plants-15-01132-s001.zip › plants-4194809-supplementary.pdf]

Supplementary Material

Impacts of Tobacco Stalk Biochar Remediation in Microplastic Contaminated Soil on Early Rice Growth Indicators and Soil Quality

Qiong Yang [1, #], Suhang Li [1, #], Rou Ma [1], Longcheng Jiang [1], Jiaojiao Liu [1], Jiaxin Yao [1], Ying Liu [2], Jun Ren [1], Yang Luo [1], Yangzhou Xiang [1, *] and Xuqiang Luo [1, *]

[1] School of Geography and Resources, Guizhou Education University, Guiyang 550018, China
[2] School of Biological Sciences, Guizhou Education University, Guiyang 550018, China
# These authors contributed equally to this work.
* Correspondence: Correspondence: yzhxiang18@126.com (Y.X.); xuqiangluo@gznc.edu.cn (X. L.)

Table S1. Results of two-way ANOVA on soil pH and organic matter (SOM) in response to TSB and PE-MPs. "PE-MPs × TSB" denotes the interaction effect between PE-MPs and TSB.

Variables	Source	Sum of Squares	F	Significance
	PE-MPs	1.083	46.211	0.000
pH	TSB	7.554	322.307	0.000
	PE-MPs × TSB	0.101	1.439	0.213
	PE-MPs	119.473	2.121	0.117
SOM	TSB	15437.890	274.080	0.000
	PE-MPs × TSB	655.131	3.877	0.002

Table S2. Results of two-way ANOVA on soil available nutrients in response to TSB and PE-MPs. "PE-MPs × TSB" denotes the interaction effect between PE-MPs and TSB.

Variables	Source	Sum of Squares	F	Significance
	PE-MPs	1113.777	184.346	0.000
SAN	TSB	334.584	55.379	0.000
	PE-MPs × TSB	86.419	4.768	0.000
	PE-MPs	5336.525	296.114	0.000
SAP	TSB	14218.364	788.951	0.000
	PE-MPs × TSB	797.181	14.745	0.000
	PE-MPs	4539.372	628.382	0.000
SAK	TSB	11730.022	1623.778	0.000
	PE-MPs × TSB	551.375	25.442	0.000

Table S3. Results of two-way ANOVA on growth parameters of rice seedling in response to TSB and PE-MPs. "PE-MPs × TSB" denotes the interaction effect between PE-MPs and TSB.

Variables	Source	Sum of Squares	F	Significance
	PE-MPs	2699.074	24.292	0.000
Germination rate	TSB	4165.741	37.492	0.000
	PE-MPs × TSB	415.741	1.247	0.303
	PE-MPs	537019.338	29.610	0.000
Vigour index	TSB	3665996.394	202.131	0.000
	PE-MPs × TSB	133877.742	2.461	0.029
	PE-MPs	2112.039	46.667	0.000
Stress tolerance index	TSB	14864.632	328.445	0.000
	PE-MPs × TSB	744.258	5.482	0.000

Table S4. Results of two-way ANOVA on physiological properties of rice seedling in response to TSB and PE-MPs. "PE-MPs × TSB" denotes the interaction effect between PE-MPs and TSB.

Variables	Source	Sum of Squares	F	Significance
	PE-MPs	3180.980	66.453	0.000
SOD	TSB	1127.905	23.563	0.000
	PE-MPs × TSB	1043.185	7.264	0.000
	PE-MPs	298.103	153.671	0.000
CAT	TSB	504.392	260.011	0.000
	PE-MPs × TSB	352.539	60.577	0.000
